# Comparison of the diagnostic value of white light endoscopic, narrow band imaging, and iodine staining individually and in combination for early esophageal cancer and precancerous lesions

**DOI:** 10.3389/fonc.2026.1810538

**Published:** 2026-07-10

**Authors:** Xia Yin, Miao Meng

**Affiliations:** Gastroenterology Department, Wuxi No.2 People’s Hospital, Wuxi, Jiangsu, China

**Keywords:** early esophageal cancer, iodine staining, narrow band imaging, precancerous lesions, white light endoscopic

## Abstract

**Objective:**

To compare the diagnostic efficacy of white light endoscopic (WLE), narrow band imaging (NBI), and iodine staining, both individually and in combination, for the detection of early esophageal cancer (EC) and precancerous lesions.

**Methods:**

A retrospective analysis was conducted on 55 patients with suspected early EC or precancerous lesions who were treated at Wuxi Second People’s Hospital between January 2022 and June 2023. All patients underwent simultaneous endoscopic examination using WLI, NBI, and iodine staining. A total of 63 lesions were identified. Surgical histopathological results and/or follow-up confirmation served as the gold standard. The diagnostic performance (including accuracy, sensitivity, and negative predictive value) of each method individually and in combination was observed and compared.

**Results:**

Among the 63 suspected lesions, 5 cases of early EC and 37 cases of precancerous lesions were confirmed by histopathology or follow-up. The combined diagnostic and iodine staining approach demonstrated significantly higher accuracy and sensitivity for early EC compared to WLI or NBI alone (*P* < 0.05). Agreement with pathological findings was poor for WLI (Kappa = 0.327), fair for NBI (Kappa = 0.476), fair for iodine staining (Kappa = 0.577), and substantial for the combined method (Kappa = 0.715). Among negative lesions, the false positive rate for mucosal staining was lower than that for background coloration.

**Conclusion:**

Both endoscopic NBI and iodine staining exhibit relatively high clinical detection rates for early EC and precancerous lesions. Their combined use can further enhance the detection rate of these conditions.

## 1 Introduction

Esophageal cancer (EC) ranks as the seventh most commonly occurring cancer and the sixth leading cause of cancer-related deaths worldwide, posing a serious threat to human health. Its occurrence rate is higher in developing countries than in developed nations (1). China accounts for more than 50% of the global burden of EC cases, with both incidence and mortality rates being higher in rural areas compared to urban regions (2). Therefore, early screening and diagnosis of EC can significantly reduce the medical burden on both the nation and individuals.

Gastrointestinal endoscopy serves as an effective method for EC screening (3). Early EC typically presents under conventional white light endoscopy (WLE) as erythema, color changes, mucosal roughness, and indistinct mucosal patterns (4). These features are non-specific, particularly in flat lesions, which are more challenging to identify, leading to high rates of underdiagnosis and misdiagnosis. Narrow band imaging (NBI) endoscopy enhances the visualization of microvascular and microstructural patterns of the esophageal mucosa, demonstrating a strong capability for detecting early EC and precancerous lesions (5). Meanwhile, endoscopic iodine staining, owing to its low cost, simplicity of operation, and high positive rate, has also shown favorable effectiveness in identifying early EC and precancerous lesions (6).

This study aims to compare the diagnostic value of WLE, NBI, and iodine staining, both individually and in combination, in the detection of early EC and precancerous lesions.

## 2 Data and methods

### 2.1 General information of the patient

This study adopts a continuous case enrollment method, clinical data of 67 patients with suspected early-stage EC and precancerous lesions who underwent endoscopic examination at our hospital between January 2022 and June 2023 were retrospectively analyzed. Inclusion criteria were as follows: (1) first-time pathological examination and surgical treatment for EC; (2) availability of complete medical records, including WLE, thoracic imaging, and pathological biopsy; (3) meeting the indications for screening of early esophageal squamous cell carcinoma and precancerous lesions (7); (4) endoscopic examination, endoscopic submucosal dissection, and pathological examination are all performed in our hospital and (5) provision of informed consent by both the patients and their families. Exclusion criteria included:(1) pregnancy or lactation; (2) iodine allergy; (3) renal disease; (4) concurrent upper gastrointestinal polyps, esophageal varices, Barrett’s esophagus, or other related lesions; and (5) gastrointestinal obstruction or perforation.

**Figure 1:**
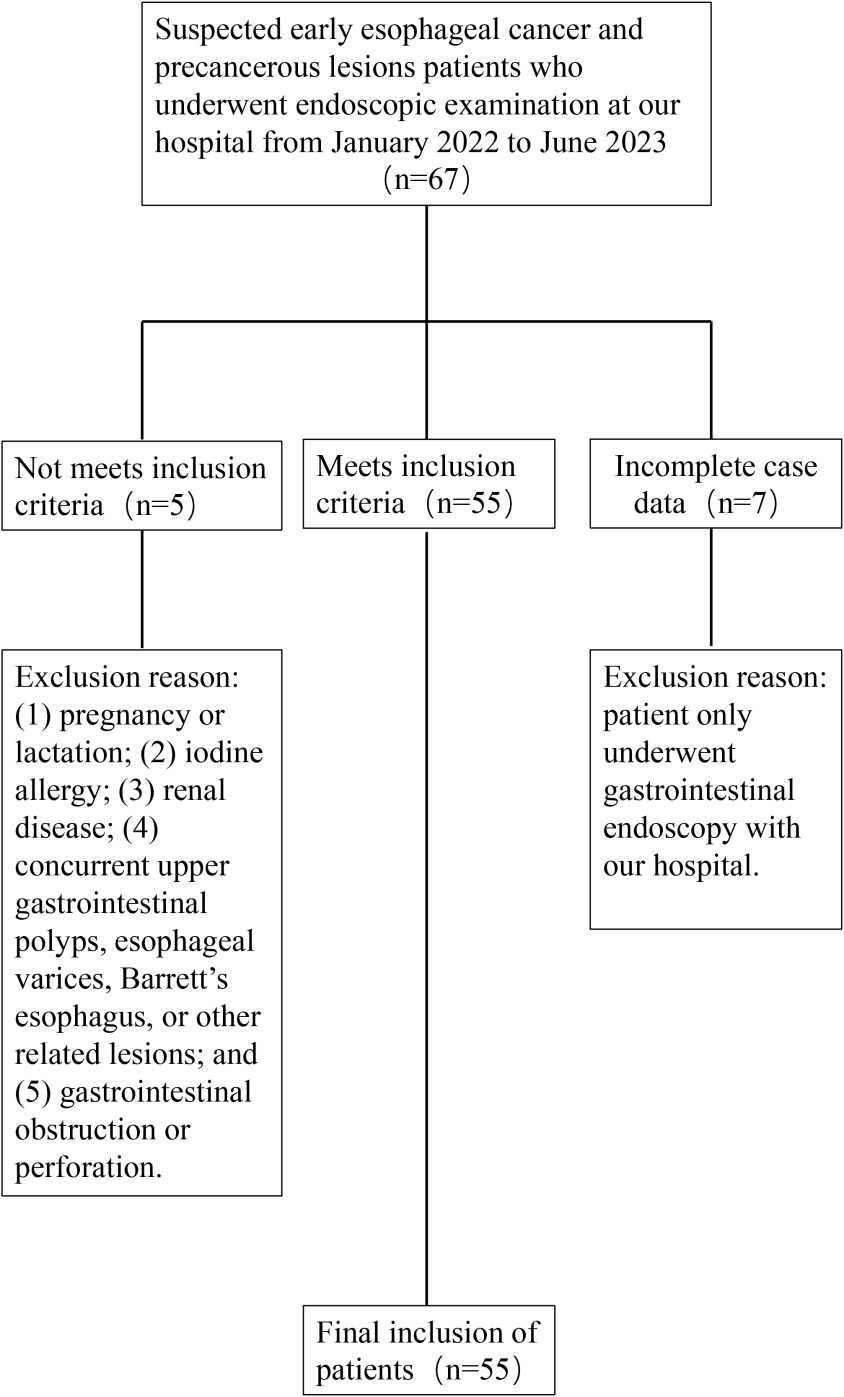
Patient screening process diagram.

A total of 55 patients were ultimately included (The inclusion process is shown in **Figure 1**), comprising 38 males and 17 females, with an age range of 46 to 88 years (mean 62.75 ± 9.52 years). A total of 63 lesions were identified, distributed as follows: upper esophagus (n = 5), middle esophagus (n = 25), and lower esophagus (n = 33) (see **Table 1**). This study was conducted in accordance with the principles of the Declaration of Helsinki.

**Table 1:** Basic clinical information of patients.

General information of patients	
Gender	
male	38
female	17
age (years)	62.75 ± 9.52
Smoking history	
yes	41
no	14
Alcohol history	
yes	36
no	19
Helicobacter pylori infection history	
yes	12
no	43
Lesion site	
cervical segment of esophagus	5
thoracic segment of esophagus	25
abdominal segment of esophagus	33

### 2.2 Method of examination

The procedures were performed using an OLYMPUS CV-260SL electronic gastrointestinal main unit and light source, along with a GIF-H290Z magnifying endoscope. All endoscopic examinations were conducted by physicians with over 10 years of experience in endoscopy. Prior to the procedure, all patients underwent routine fasting and received pharyngeal anesthesia.

WLI endoscopy: The endoscope was inserted slowly through the oropharynx and advanced through the upper esophageal sphincter into the esophageal lumen. An adequate amount of air was immediately insufflated to fully distend the esophageal wall and avoid obscuring lesions by mucosal folds. The endoscope was advanced gradually along the esophageal wall while the entire esophagus was examined systematically and continuously. All areas including the anterior, posterior, left, and right walls were carefully inspected to avoid missing any lesions. After reaching the esophagogastric junction, the endoscope was slowly withdrawn. If mucus, foam, or food residue was encountered, the mucosal surface was irrigated using the water-jet function and subsequently suctioned to maintain a clear visual field. Simultaneously, the bending section and rotation of the endoscope were adjusted to visualize behind and alongside each fold, ensuring no blind areas remained. Magnifying endoscopy with NBI (ME-NBI): When suspicious lesions were detected under WLI or when detailed inspection of the entire esophagus was required, the NBI mode was activated by pressing the NBI button on the endoscope handle or main unit to switch from white light to NBI. If the visual field was unclear after switching, water was immediately delivered to rinse the mucosal surface. Iodine staining: When a suspicious lesion was identified under WLI, approximately 15–20 mL of 1.5% Lugol’s iodine solution was sprayed thoroughly over the lesion and surrounding mucosa. Staining results were observed and recorded 2–3 minutes after application. Three doctors analyzed the WLE, NBI, and iodine staining images separately.

All patients underwent endoscopic submucosal dissection. Postoperative pathological diagnosis was used as the gold standard for final diagnosis.

### 2.3 Observation indicators and standard of criterion

The pathological findings and the discrepancies among the three detection methods were analyzed, and the diagnostic concordance rate was calculated. Low-grade intraepithelial neoplasia (LGIN) included mild and moderate esophageal dysplasia, while high-grade intraepithelial neoplasia (HGIN) included severe dysplasia and carcinoma *in situ*. Early EC was defined as intramucosal carcinoma or submucosal carcinoma without lymph node metastasis. Precancerous lesions include LGIN and HGIN, while early EC and HGIN are considered pathologically positive.

Under WLE and NBI, the size, number, boundary clarity, and presence of background coloration of the lesions were recorded. After iodine staining, the stained positive areas, number of lesions, and boundary clarity were documented.

For WLE and NBI, positivity was defined as abnormal esophageal mucosal coloration, surface roughness, erosion or elevation, unclear boundaries, and brownish discoloration. For iodine staining, positivity was defined as faint staining (grade III) or no staining (grade IV), while dark staining (grade I) and normal staining (grade II) were considered negative. If any one of the above three results is positive, the combined examination will be positive.

### 2.4 Statistical methods

Statistical analysis was performed using SPSS 21.0. Categorical data were expressed as n (%), and intergroup comparisons were conducted using the *χ²* test. The normality of continuous data was assessed using the Shapiro–Wilk test. Data conforming to a normal distribution were presented as mean ± standard deviation, while non-normally distributed data were described using interquartile ranges. Intergroup comparisons were performed using the *t*-test. The accuracy, sensitivity, specificity, positive predictive value, and negative predictive value of the inspection method are tested using paired data McNemar test. A Kappa value ≥ 0.75 indicated high agreement, a value between 0.40 and 0.74 indicated moderate agreement, and a value < 0.40 indicated poor agreement. A *P*-value < 0.05 was considered statistically significant.

## 3 Results

### 3.1 Comparison of the diagnostic efficacy of WLE, NBI, and iodine staining individually and in combination

**Figure 2:**
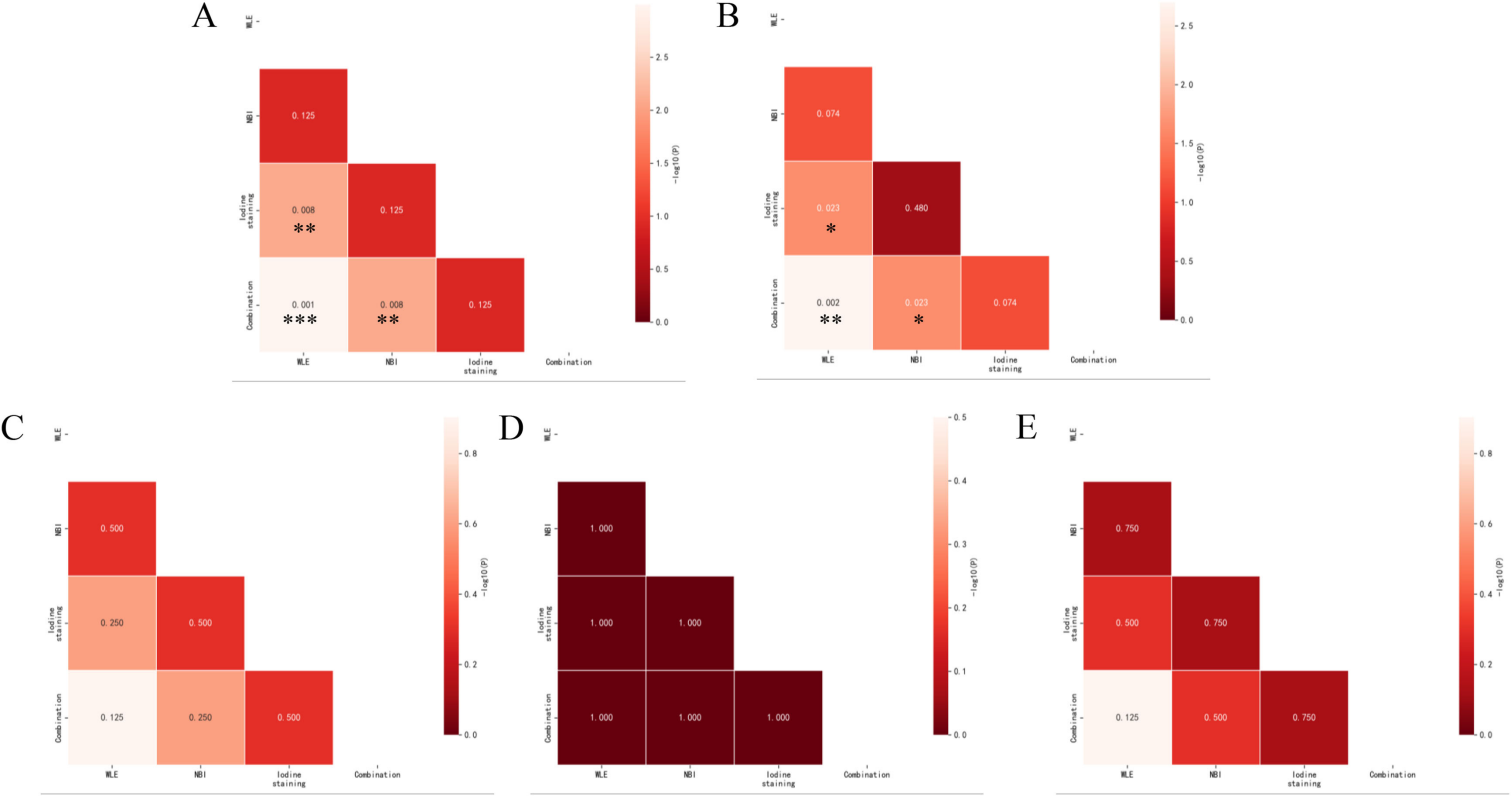
The McNemar test analyzes the direct differences in accuracy, sensitivity, specificity, positive predictive value, and negative predictive value among different examination methods. **(A)** Comparison of accuracy of different inspection methods. **(B)** Comparison of sensitivity of different inspection methods. **(C)** Comparison of specificity of different inspection methods. **(D)** Comparison of positive predictive value of different inspection methods. **(E)** Comparison of negative predictive value of different inspection methods. *<0.001, **<0.01, ***<0.05.

The accuracy of combined examination is higher than WLE and NBI, and the accuracy of iodine staining is higher than WLE. At the same time, the sensitivity of combined examination is higher than WLE and NBI, and the sensitivity of iodine staining is higher than WLE. (*P* < 0.05). There was no statistically significant difference in the specificity, positive predictive value, and negative predictive value between WLE、NBI、iodine staining and combined examination. (*P* > 0.05). See **Table 2**; **Figure 2**.

**Table 2:** Comparison of diagnostic value of three detection methods alone and in combination (%, 95% CI).

Inspection method	Accuracy	Sensitivity	Specificity	Positive predictive value	Negative predictive value
WLE	68.25, 55.98–78.45	48.15 (29.84–66.97)	83.33 (67.58–92.70)	68.42 (45.64–85.06)	68.18 (53.01–80.46)
NBI	74.60 (62.59–83.88)	66.67 (47.15–82.02)	80.56 (64.62–90.56)	72.00 (51.78–86.09)	76.32 (60.61–87.37)
iodine staining	79.37 (67.78–87.56)	74.07 (54.63–87.27)	83.33 (67.58–92.70)	76.92 (57.39–89.44)	81.08 (65.45–90.97)
combination	85.71 (75.01–92.42)	92.59 (76.00–98.12)	80.56 (64.62–90.56)	78.13 (60.95–89.13)	93.55 (78.57–98.52)

WLE: white light endoscopy, NBI: narrow band imaging.

### 3.2 Consistency analysis of individual and combined diagnostic performance of WLE, NBI, and iodine staining

Pathological diagnosis identified 27 positive and 36 negative lesions out of 63 examined sites. WLE detected 19 positive and 44 negative cases, demonstrating poor agreement with pathological findings (Kappa = 0.327,95% CI, 0.083-0.571). NBI identified 25 positive and 38 negative cases, showing moderate agreement with pathological results (Kappa = 0.476,95% CI, 0.254-0.698). Iodine staining detected 26 positive and 37 negative cases, also indicating moderate agreement with pathology (Kappa = 0.577,95% CI, 0.372-0.782). In contrast, the combined diagnostic approach identified 32 positive and 31 negative cases, exhibiting substantial agreement with pathological diagnosis (Kappa = 0.715,95% CI, 0.543-0.887), as summarized in **Table 3**.

**Table 3:** Comparison of individual and combined diagnostic methods with pathological findings.

Inspection method		Pathological results		Total
		Positive	Negative	
WLE	positive	13	6	19
	negative	14	30	44
NBI	positive	18	7	25
	negative	9	29	38
iodine staining	positive	20	6	26
	negative	7	30	37
combination	positive	25	7	32
negative	2	29	31
total		27	36	63

WLE: white light endoscopy, NBI: narrow band imaging.

### 3.3 Relationship between background coloration with NBI and mucosal staining grade with iodine staining in different lesions

**Figure 3:**
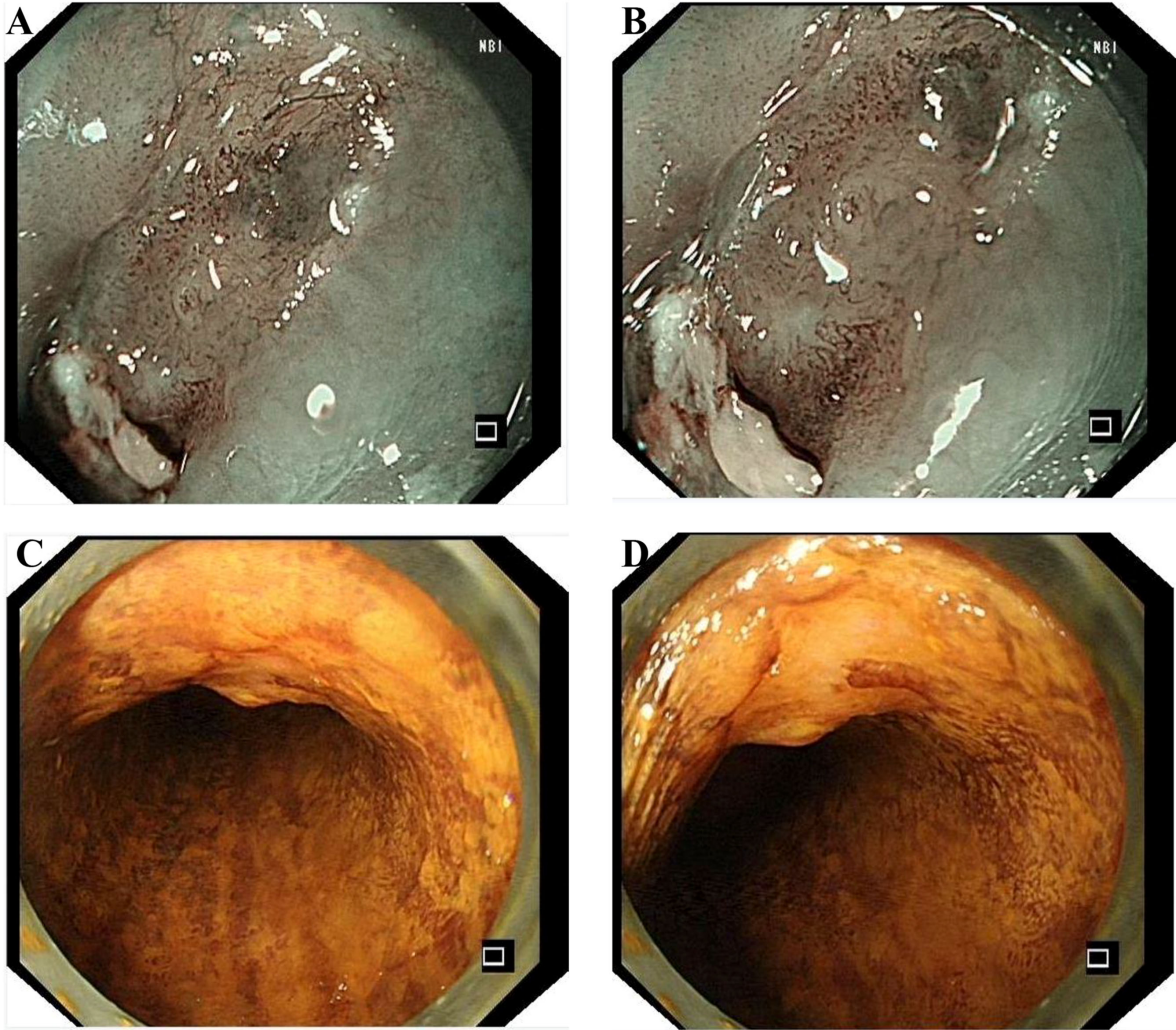
Typical case. Male, 77 years old, admitted to the hospital with the main complaint of choking while eating, the final diagnosis was squamous cell carcinoma. **(A, B)** NBI findings: The microvascular architecture on the mucosal surface appears disorganized and dense, with punctate dilatation, varying calibers, and irregular shapes, accompanied by a positive background coloration sign. **(C, D)** Iodine staining findings: The mucosa exhibits lack of staining.

Pathological results revealed that among the 63 lesions, 5 were early EC, 22 were HGIN, 15 were LGIN, 18 were inflammatory lesions, and 3 were normal mucosa. Among the pathologically positive results (early EC and HGIN), both the background coloration sign (92.59%, 25/27) and mucosal staining (92.59%, 25/27) exhibited high positive rates (Typical cases are shown in **Figure 3**). In contrast, among the pathologically negative results (LGIN, inflammatory lesions, and normal mucosa), the false positive rate of the background coloration sign (33.33%, 12/36) was significantly higher than that of mucosal staining (5.56%, 2/36), and the difference was statistically significant (p < 0.05). Details are shown in **Table 4**.

**Table 4:** Relationship between endoscopic manifestations and pathology of the lesions.

Pathological results	Background coloration		Mucosal staining			
	Positive	Negative	I	II	III	IV
early EC	5	0	0	0	0	5
HGIN	20	2	0	2	10	10
LGIN	5	10	3	10	2	0
inflammatory lesions	7	11	2	16	0	0
normal mucosa	0	3	2	1	0	0

EC, esophageal cancer; HGIN, high-grade intraepithelial neoplasia; LGIN, low-grade intraepithelial neoplasia.

## 4 Discussion

Early EC is a relatively common type of early-stage gastrointestinal tract tumor. Early diagnosis and treatment can effectively prolong patient survival and improve quality of life (8, 9). Previously, WLE was commonly used as a standard technique for the differential diagnosis of early gastrointestinal tumors. During the examination, the vascular patterns, color, and morphology of the gastrointestinal mucosa are carefully observed under conventional light, and the extent of disease progression is assessed based on corresponding changes. However, clinical practice has revealed that WLE has limited ability to distinguish between normal and lesional mucosa, leading to a high rate of underdiagnosis (10, 11).

With advances in clinical diagnostic techniques, confocal endoscopy, chromoendoscopy, magnifying endoscopy, NBI, and iodine staining have been gradually introduced into clinical use. Under conventional WLE, early EC and its precancerous lesions may exhibit subtle mucosal changes. The esophageal mucosa often appears light red with poor contrast compared to surrounding normal tissue, limiting the effectiveness of standard endoscopy alone (12). NBI utilizes optical filters to narrow the wavelength of white light from a xenon lamp, allowing red, green, and blue light to penetrate only the superficial mucosal layer. This enhances the contrast between abnormal and normal mucosa and improves the visualization of capillaries, thereby increasing the detection rate of early superficial cancers (13, 14). ME-NBI enables detailed observation of the intrapapillary capillary loops intraepillary capillary and mucosal microstructure within the esophageal epithelium, facilitating better discrimination between lesional and normal mucosa. This approach aids in the early detection of minute esophageal mucosal abnormalities and improves the identification of precancerous lesions and early EC (15). Meanwhile, a graphic conversion algorithm called VIS-HSI and SAVE image storage technology can convert WLE images into NBI like images, which may provide more effective assistance for future EC examinations, but further validation is still needed (16, 17).

Iodine solution is an absorptive dye commonly used for esophageal staining. Its mechanism involves a color reaction when iodine binds to glycogen. Normal esophageal squamous epithelial cells are rich in glycogen and stain brownish-yellow after iodine application. In contrast, abnormal squamous epithelial cells contain reduced or absent glycogen and appear lightly stained or unstained. This color variation helps identify suspicious lesions (18). However, inter-observer variability in color interpretation after iodine staining and the risk of iodine allergy in some patients limit its widespread application (19, 20).

In this study, the combined diagnostic approach demonstrated higher accuracy, sensitivity, and negative predictive value in diagnosing early EC compared to WLE or NBI alone, and the differences were statistically significant (*P* < 0.05). However, no significant differences were observed in specificity or positive predictive value (*P* > 0.05). Moreover, the combined method showed better diagnostic consistency than any of the three techniques used individually. Among pathologically confirmed positive lesions, both background coloration and mucosal staining exhibited high positive rates. However, among pathologically negative lesions, background coloration had a higher false-positive rate than mucosal staining (*P* < 0.05).

This study has several limitations: (1) its retrospective nature may have introduced selection bias; (2) the sample size was relatively small, which may lead to insufficient efficacy in subgroup analysis and potential selection bias; (3) the extrapolation of conclusions is limited due to the highly selective patient population undergoing endoscopic submucosal dissection as the research subject. Future large-scale, multi-center studies are needed to validate these findings.

In conclusion, both NBI and iodine staining demonstrate relatively high clinical detection rates for early EC and precancerous lesions. Their combined use further enhances the detection rate of early EC and its precursor conditions.

## Data Availability

The original contributions presented in the study are included in the article/supplementary material. Further inquiries can be directed to the corresponding author.
